# Prenatal Metal Exposures and Associations with Kidney Injury Biomarkers in Children

**DOI:** 10.3390/toxics10110692

**Published:** 2022-11-16

**Authors:** Maria D. Politis, Meizhen Yao, Chris Gennings, Marcela Tamayo-Ortiz, Damaskini Valvi, Seunghee Kim-Schulze, Jingjing Qi, Chitra Amarasiriwardena, Ivan Pantic, Mari Cruz Tolentino, Guadalupe Estrada-Gutierrez, Jason H. Greenberg, Martha M. Téllez-Rojo, Robert O. Wright, Alison P. Sanders, Maria José Rosa

**Affiliations:** 1 Department of Environmental Medicine and Public Health, Icahn School of Medicine at Mount Sinai, New York, NY 10029, USA; 2 Occupational Health Research Unit, Mexican Social Security Institute, Mexico City 06600, Mexico; 3 Human Immune Monitoring Center, Icahn School of Medicine at Mount Sinai, New York, NY 10029, USA; 4 Department of Oncological Science, Icahn School of Medicine at Mount Sinai, New York, NY 10029, USA; 5 Center for Nutrition and Health Research, National Institute of Public Health, Cuernavaca 62100, Mexico; 6 Department of Developmental Neurobiology, National Institute of Perinatology, Mexico City 06600, Mexico; 7 Department of Nutrition, National Institute of Perinatology, Mexico City 06600, Mexico; 8 Research Direction, National Institute of Perinatology, Mexico City 06600, Mexico; 9 Department of Pediatrics, Section of Nephrology, Yale University School of Medicine, New Haven, CT 06510, USA; 10 Department of Pediatrics, Icahn School of Medicine at Mount Sinai, New York, NY 10029, USA; 11 Department of Environmental and Occupational Health, University of Pittsburgh, Pittsburgh, PA 15260, USA

**Keywords:** heavy metals, renal health, mixture, arsenic, cadmium, lead, mercury

## Abstract

Prenatal exposure to arsenic (As), cadmium (Cd), mercury (Hg), and lead (Pb) may be nephrotoxic, yet limited studies have examined subclinical kidney injury biomarkers in children. We assessed whether metal exposure in the second trimester (2T), a crucial time of kidney development, is associated with altered urine kidney injury and function biomarkers in preadolescent children. Analyses included 494 children participating in a birth cohort study in Mexico City. Concentrations of As, Cd, and Pb were measured from pregnant women in 2T blood and urine, and Hg in urine only. Kidney biomarkers were measured from children in urine at age 8–12 years. We assessed the associations between individual metals and (1) kidney biomarkers using linear regression and (2) a multi-protein kidney mixture using weighted quantile sum (WQS) regression. Associations of separate urine and blood metal mixtures with individual kidney biomarkers were assessed via WQS. Within the multi-protein mixture, the association with increased urinary As was predominated by urine alpha-1-microglobulin (A1M), interferon gamma-induced protein 10 (IP10), and fatty acid binding protein 1; the association with increased urinary Cd was predominated by A1M, clusterin, and albumin. The urine metal mixture was associated with increased albumin (0.23 ng/mL; 95% confidence interval (CI): 0.10, 0.37), IP10 (0.15 ng/mL; 95% CI: 0.02, 0.28), and cystatin C (0.17 ng/mL; 95% CI: 0.04, 0.31); these associations were mainly driven by urinary As and Cd. We observed null associations between prenatal blood or urine metal mixtures and estimated glomerular filtration rate. Higher prenatal urinary metals, individually and as a mixture were associated with altered kidney injury biomarkers in children. Further research and longer participant follow-up are required to ascertain the risk of kidney disease later in life.

## 1. Introduction

The prenatal period is an important stage of human development that is susceptible to toxic environmental exposures, including toxic metals and metalloids [1]. During pregnancy, metal(loid)s, such as lead (Pb), mercury (Hg), arsenic (As), and to a limited extent cadmium (Cd), can cross the placental barrier resulting in fetal exposure [2,3,4]. Metal exposure can occur through diet and drinking water as well as from anthropogenic sources including cigarette smoke, fertilizers, industrial emissions, as well as occupational sources [5,6]. In Mexico City, the primary sources of Pb exposure include air pollution and diet, partially through the use of traditional Pb-glazed ceramics used to prepare and store food [7,8]. Exposure to metal(loid)s during the prenatal period is associated with adverse birth outcomes and poorer childhood health, including low birthweight, risk of adverse respiratory symptoms, and higher blood pressure [9,10,11]. Additionally, metal(loid)s are nephrotoxic elements that can affect kidney development altering glomerular or tubular function, which are vital for regulation of blood pressure, excretion of drugs, and maintenance of electrolytes, water, toxicants, and nutrient homeostasis, among other functions [12].

Increasing severity of kidney damage, assessed by markers of tubular injury such as increased urinary neutrophil gelatinase-associated lipocalin (NGAL) [13], and increased urinary kidney injury molecule-1 (KIM-1) [14], are associated with a greater risk of chronic kidney disease (CKD) and end stage renal disease [15,16]. Additionally, markers that are freely filtered by the glomerulus such as serum cystatin C and serum creatinine can be used to assess kidney function [17]. Urinary kidney injury biomarkers, which include proteins secreted by the tubules such as beta-2-microglobulin (B2M), or that escape the glomerular filtration barrier such as albumin, provide sensitive indicators of kidney injury and dysfunction that can occur in the presence or absence of a rise in traditional clinical markers such as serum creatinine [18]. Assessment of urinary proteins such as NGAL, B2M, KIM-1, albumin, and their combinations, offer improved sensitivity compared to traditional indicators in diagnosing kidney injury and have been suggested to improve the prognosis of CKD [18,19]. Further, these protein biomarkers can be collected non-invasively in the urine, and may better detect nephrotoxic insults, including to metals such as As, Pb, and chromium [20,21,22].

Several studies have examined the association between environmental exposures with pediatric kidney function and kidney disease [20,23,24,25,26]. However, there is a paucity of research on in utero exposure to metals and metalloids, a time of dramatic growth and development when exposure may have a severe and long-standing impact on kidney function. Exposure to metals, including when assessed as a mixture, has demonstrated moderate positive associations with traditional indicators of pediatric kidney function such as estimated glomerular filtration rate (eGFR) [27]. Similar findings in animal studies showed that metal mixtures in drinking water (including As, Cd, vanadium, and Pb), even at low environmental levels impaired kidney development in zebrafish embryos at early stages of pronephros development, a developmental window comparable to ~3–4 weeks’ gestation in the human fetus [12,28]. In the human fetus, metanephric kidney development (formation of the permanent functional kidney) is initiated at 4 to 5 weeks’ gestation to the beginning of the second trimester, with nephrogenesis and the progression of tubular functions occurring from 6 to 36 weeks’ gestation, with nephrons continuing to expand and mature beyond 36 weeks [29,30]. The impact of environmental factors during this period may lead to a reduced nephron number, increasing the risk of kidney disease in later life [31]; therefore, due to the timing of kidney and nephron development in utero, and evidence in prior work [32,33,34], we selected the second trimester to examine potential susceptibility to metal exposure. This study aimed to assess the associations between prenatal metal exposure during the second pregnancy trimester, a relevant window of kidney development, with novel urinary kidney injury biomarkers and eGFR assessed at preadolescence (ages 8–12 years).

## 2. Materials and Methods

### 2.1. Study Population

**Figure 1:**
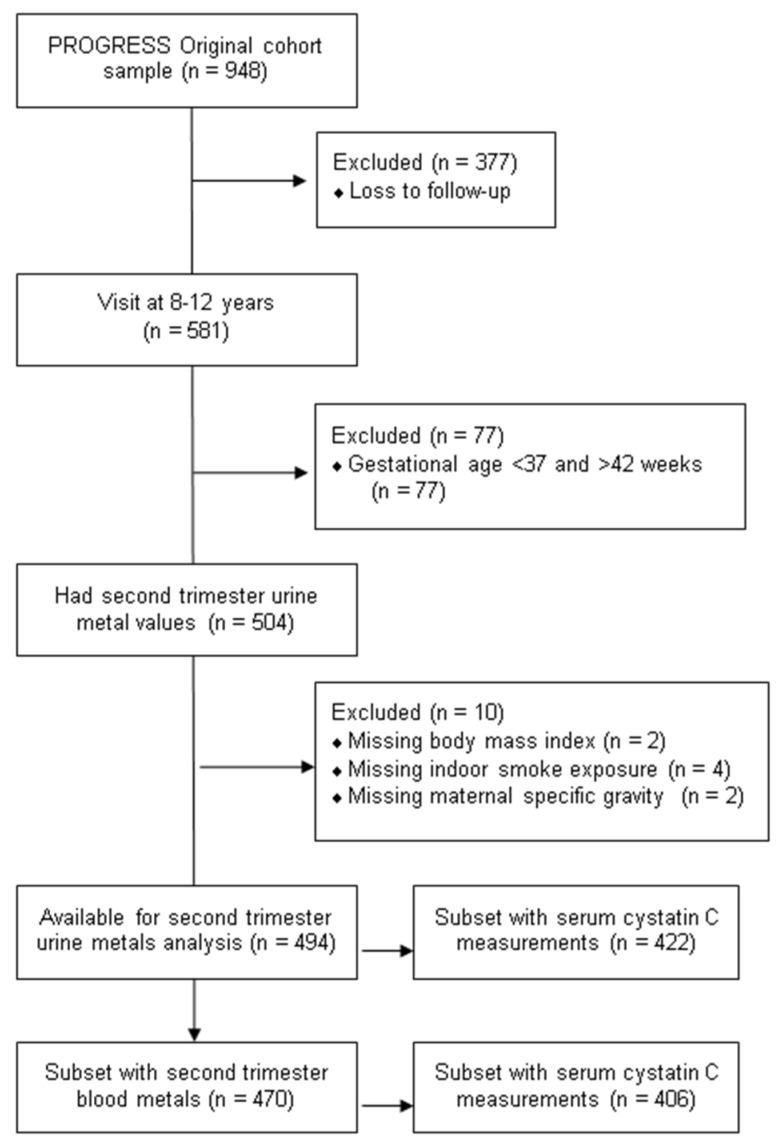
Flowchart of participant selection and analytic subsets.

This longitudinal analysis included data from the Programming Research in Obesity, Growth, Environment and Social Stressors (PROGRESS) study, a birth cohort based in Mexico City. Between 2007 and 2011, pregnant women in their second trimester were recruited through the Mexican Social Security Institute (Instituto Mexicano del Seguro Social). Women were enrolled if they were at least 18 years of age, less than 20 weeks’ gestation, no medical history of heart or kidney disease, no daily alcohol consumption, and no use of steroids or anti-epilepsy drugs. A total of 948 women delivered a live child into the cohort, and 581 children attended the 8–12-year visit. We excluded participants who had missing BMI (n = 2), indoor smoke exposure during second trimester (n = 4), second trimester urine specific gravity data (n = 4) and had a gestational age of less than 37 weeks and greater than 42 weeks (n = 67). The final sample size was 494 mother–child dyads with available prenatal urine metals and children’s protein measurements (Figure 1). In secondary analyses, we examined the relationships in 470 mother–child dyads with available blood metals measured in the second trimester. Serum cystatin C was measured for 422 children with available prenatal urine metals and for 406 children with available prenatal blood metals; in analyses with eGFR as the outcome, the sample size was further restricted to these subsets.

As of the 8–12-year study visit, children were generally healthy and free of cardiovascular or kidney disease, assessed through a maternal questionnaire. All data collection methods were conducted in accordance with the appropriate regulations and guidelines, and written informed consent was obtained from the mothers prior to the collection of samples, and children’s assent was obtained at the 8–12-year visit. The study protocols for PROGRESS were approved by the institutional review boards (IRB) of the Icahn School of Medicine at Mount Sinai (IRB protocol number: 12-00751A), Brigham and Women’s Hospital, and the National Institute of Public Health in Mexico.

### 2.2. Second Trimester Metals Assessment

Blood and urine samples were collected from pregnant women to measure metal exposure in the second trimester of pregnancy. Blood samples were stored at 4 °C, urine samples were stored at −80 °C, and they were shipped to the Icahn School of Medicine at Mount Sinai for subsequent metal analysis [35]. As previously reported [36], blood and urine samples were digested in 0.5% HNO_3_, 0.005% Triton X-100, and mixed with an internal standard and analyzed on an Agilent 8900 ICP Triple Quad mass spectrometer (ICP-QQQ) (Agilent Technologies, Inc., Santa Clara, CA, USA) in tandem mass spectrometry (MS/MS) mode with cell gases to eliminate molecular ion interferences using matrix-matched calibration standards. To correct for differences in sample introduction, ionization, and reaction rates, internal standards (tellurium for As, rhodium for Cd, and lutetium for Pb) were used, as well as in-house pooled urine or blood samples to monitor for accuracy and reproducibility for each analytic batch. Quality control measures were as previously described [27,37].

Urine specific gravity was measured using a Rudolph J157HA+ Automatic Refractometer (Rudolph Research, New Jersey). The following formula was used to correct for the hydration status of second trimester urine metal concentrations:

(1) $$Metal_{Corrected}=Metal_{Original}\;\times \;\frac{\mu_{SG}-1}{SG-1}.$$

$Metal_{Corrected}$ is the corrected metal concentration, $Metal_{Original}$ is the original metal concentration, *µ_SG_* is the mean specific gravity value (1.02 for the second trimester samples in this study), and *SG* is the specific gravity.

### 2.3. Child Urinary Protein Biomarkers and Urine Creatinine

Spot urine samples were collected from children at the 8–12-year visit. Urinary protein concentrations were assessed at the Mount Sinai Human Immune Monitoring Core by three human acute kidney injury multiplex panels that included 17 proteins with established or putative evidence with kidney injury in prior studies. Multiplexed enzyme-linked immunoassays (ELISA) were performed using the Luminex-200 multiplex system to quantify protein concentrations using Milliplex xMAP technology (EMD Millipore, Billerica, MA, USA). Panel 1 included calbindin, glutathione S-transferase alpha (GSTα), TIMP metallopeptidase inhibitor 1 (TIMP1), KIM-1, interferon gamma-induced protein 10 (IP10), renin, and fatty acid binding protein 1 (FABP1). Panel 2 included epidermal growth factor (EGF), NGAL, albumin, clusterin, cystatin C, osteopontin (OPN), and alpha-1-microglobulin (A1M). Panel 3 included uromodulin, retinol-binding protein 4 (RBP4), and B2M. Mean fluorescence intensity (MFI) values were measured for each analyte and converted to absolute quantitation levels based on linear internal standard curves. Normalization for each batch was completed using two quality control reference standards according to the manufacturer’s instructions as well as normalized using a normal healthy donor pooled urine. The absolute quantification values after normalization for each protein were used in subsequent analyses. Protein concentrations that were below the lower limit of detection (LOD) were replaced with the value of LOD divided by the square root of two. Protein concentrations above the quantifiable range were excluded from analyses. This included albumin (n = 3), NGAL (n = 1), OPN (n = 1), and B2M (n = 1). Nearly 36% (n = 176) of uromodulin MFI values were above the quantifiable range; thus, we performed exploratory analyses using log_2_ transformed uromodulin MFI values. The protein biomarkers were grouped by glomerular, tubular segment-specific, liver, or general (non-specifically expressed) proteins, based on their sites of expression and the pathophysiologic mechanisms that correspond to clinical acute kidney injury [38,39]. Glomerular proteins included albumin and cystatin C, tubular proteins included KIM-1, NGAL, A1M, B2M, RBP4, OPN, uromodulin, and GSTα. Liver proteins included FABP1 and ‘general’ proteins included EGF, clusterin, calbindin, TIMP1, IP10, and renin. Children’s urine creatinine measurements were conducted using Arbor Assay’s Urine Creatinine Detection Kit, and all samples were diluted with water at 1:100 dilution and pipetted into a 96-microwell plate with creatinine reagent for analysis on a SpectraMax Plus 385 plate reader (Molecular Devices, San Jose, CA, USA).

### 2.4. Serum Cystatin C and eGFR

The Quantikine^®^ human cystatin C immunoassay (R&D Systems, Minneapolis, MN, USA) was used to obtain the measurements of serum cystatin C. Using the cystatin C measurements, the eGFR values were derived using the following formula:

eGFR = 70.69 × (cystatin C)^−0.931^, (2)

where cystatin C is in mg/L [40].

### 2.5. Covariates

Additional information was collected from participants through questionnaires, including child age, sex, maternal report of prenatal indoor smoke exposure, and socioeconomic status (SES) during pregnancy. Child body mass index (BMI) was measured at the same time as the collection of urine for the kidney injury proteins and the estimation of age- and sex-specific BMI *z*-scores was based on the World Health Organization Growth Reference [41]. BMI was categorized into 3 levels: normal weight (BMI *z*-score ≤ 1), overweight (1 < BMI *z*-score ≤ 2), and obese (BMI *z*-score > 2). Indoor smoke exposure during pregnancy was defined as a report of any smoker in the home during the second or third trimester. Individual level SES was assessed utilizing 13 variables derived from prenatal questionnaire results which were used to classify study participant families into six levels based on the SES index created by the Asociación Mexicana de Agencias de Investigación de Mercados y Opinión Pública (AMAI) [42]. These levels were then collapsed into lower, medium, and higher SES.

### 2.6. Statistical Analyses

All protein concentrations and urine and blood metals had non-normal residuals and therefore were log_2_ transformed. We preformed linear regression models to assess the association between individual second trimester urinary and blood metals measured in pregnant women and individual urinary biomarkers and eGFR measured in children at age 8–12 years in separate models. Covariates in adjusted models included child age (years), sex, SES (lower, medium, higher), child BMI (continuous *z*-score), prenatal indoor smoke exposure (yes/no), and child urine creatinine to account for urinary dilution, selected according to the prior literature. As a secondary analysis, we preformed the same linear regression models with metal concentrations grouped into quartiles and the log_2_ transformed kidney injury biomarker concentrations, adjusted for the covariates listed above. These regression models were preliminary analyses to inform the repeated holdout weighted quantile sum (WQS) regressions, described below [43].

We decided to use WQS for our analyses to examine the mixture effect and to observe how all of the components performed jointly on an outcome, as well as accounting for auto-correlation among the data. WQS was used in two ways: (1) to assess individual metals with a multi-protein kidney mixture and (2) to assess individual kidney injury biomarkers with separate urine and blood metal mixtures. Individual metal predictors were divided into quartiles to assess the multi-protein kidney mixture and individual kidney injury biomarkers were divided into deciles to assess metal mixtures. We constrained the directionality of the WQS models in both the positive and negative directions. Our final WQS model included weights that were the mean weight across 100 bootstrapped datasets and constrained to be both non-negative and sum to one. All models were adjusted for child age, child sex, child urine creatinine, indoor tobacco smoke exposure, SES, and BMI. We adjusted for child urine creatinine as a covariate rather than directly normalizing the urine kidney biomarkers because the urine kidney biomarker outcomes were measured in urine. Normalizing urine protein levels directly by urine creatinine may not be appropriate as urine creatinine can indicate glomerular dysfunction, as well as be associated with certain sociodemographic factors (including age, BMI, and sex) and urine outcomes [44,45]. Analyses were conducted using SAS v9.4 (SAS Corporation, Cary, NC, USA) and R Version 4.0.3 (R Development Core Team, Vienna, Austria).

## 3. Results

### 3.1. Characteristics of the Study Population

Table 1 displays the sociodemographic characteristics of the study population. The average age of the children in this study was 9.66 years (standard deviation ±0.69). Males and females were equally distributed and over half of the children (55%) were normal weight, and 24% and 21% were classified as with overweight and obesity, respectively. Less than a third of the mothers reported prenatal exposure to indoor tobacco smoke in the home. Four participants had an eGFR less than 60 mL/min/1.73 m^2^, a level potentially indicative of CKD in adults [46]. The median values of specific gravity normalized prenatal urinary As, Cd, Pb, and Hg in this study were 13.72, 0.22, 3.42, and 1.12 μg/L, respectively. Among prenatal blood metals, the median value for As was 0.07 μg/dL, 0.02 μg/dL for Cd, and 2.85 μg/dL for Pb. The Pearson correlation matrix including the urine and blood metals, and the kidney biomarkers are displayed in Supplemental Figure S1. We also report kidney injury biomarker concentrations normalized by urine creatinine, shown in Supplemental Table S1.

### 3.2. Pairwise Associations of Individual Metals with Individual Kidney Injury Biomarkers

Results of the single metal linear regression predicting eGFR and individual urinary kidney biomarker concentrations are shown in Table 2. We observed specific pairwise metal associations with proteins including albumin, cystatin C, KIM-1, A1M, B2M, EGF, clusterin, TIMP1, and IP10. No significant associations were observed between prenatal blood or urine metals and eGFR. In single metal analyses, a doubling of urine Cd was associated with 0.22 ng/mL (95% Confidence Interval (CI): 0.11, 0.33) higher albumin, 0.13 ng/mL (95% CI: 0.03, 0.23) higher cystatin C, 0.08 ng/mL (95% CI: 0.02, 0.14) higher A1M, 0.14 ng/mL (95% CI: 0.01, 0.28) higher B2M, 0.07 ng/mL (95% CI: 0.01, 0.12) higher EGF, 0.12 ng/mL (95% CI: 0.03, 0.22) higher clusterin, and 0.10 ng/mL (95% CI: 0.04, 0.16) higher TIMP1. A doubling of urine Pb was associated with 0.08 ng/mL (95% CI: 0.01, 0.16) higher KIM-1 and 0.06 ng/mL (95% CI: 0.005, 0.11) higher TIMP1. Higher albumin levels were associated with three urine metals (As, Cd, and Pb). A doubling of blood As was associated with −0.25 ng/mL (95% CI: −0.50, −0.001) lower B2M, −0.28 ng/mL (95% CI: −0.49, −0.07) lower RBP4, and −0.11 ng/mL (95% CI: −0.22, −0.003) lower EGF. In the MFI-based analysis of uromodulin, a doubling of blood As was associated with −0.18 (95% CI: −0.34, −0.02) lower uromodulin. A doubling of blood Cd was associated with 0.47 ng/mL (95% CI: 0.09, 0.85) higher GSTα. We observed null associations between blood levels of Pb with all assessed kidney injury proteins. As a secondary analysis, the results of the single metal linear regression models with metal concentrations as quartiles and kidney injury biomarker concentrations for comparison with the results of the WQS regression models, are shown in Supplemental Table S2.

### 3.3. Associations of Individual Metals with Multi-Protein Mixture

**Figure 2:**
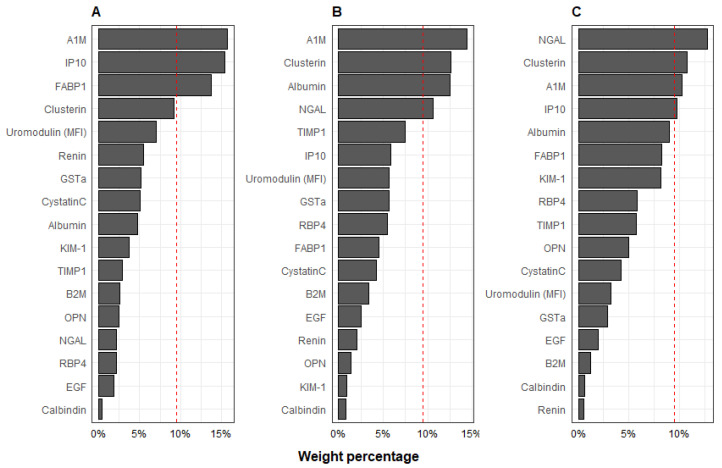
Protein weights derived in WQS regression analyses with second trimester urine (**A**) As, (**B**) Cd, and (**C**) Pb and the multi-protein mixture. Models shown were constrained in the positive direction with 100 repeated holdout validation, adjusted for urinary creatinine, socioeconomic status, child age, sex, smoking inside home, and body mass index *z*-score.

Results of individual metal associations with a multi-protein mixture are shown in Figure 2. The WQS constrained in both the positive and negative direction resulted in positive beta estimates; therefore, we constrained the WQS in the positive direction and present those results. The multi-protein indices were associated with increased urinary As, Cd, and Pb. Per each decile increase in the urine multi-protein mixture, urinary As was 0.15 µg/L (95% CI: 0.05, 0.25) higher. The protein contributions associated with increased urinary As levels were predominated by A1M (16%), IP10 (15%), and FABP1 (14%). Per each decile increase in the urine multi-protein mixture, urinary Cd was 0.12 µg/L (95% CI: 0.04, 0.20) higher. Within the multi-protein mixture associated with increased urinary Cd, the index weights were predominated by A1M (14%), clusterin (13%), albumin (12%), and NGAL (11%). Urinary Pb was 0.10 µg/L (95% CI: 0.004, 0.20) higher in the urine multi-protein mixture with protein contributions predominated by NGAL (13%), clusterin (11%), A1M (10%), and IP10 (10%).

### 3.4. Associations of Individual Kidney Injury Biomarkers with Metal Mixture Index

**Figure 3:**
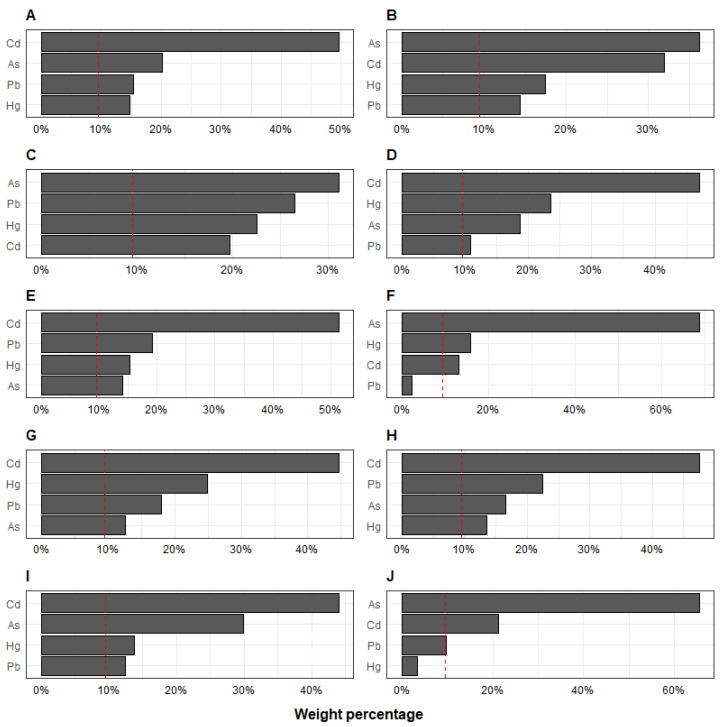
Second trimester urine metal weights derived for the multi-metal index in weighted quantile sum regression analyses with individual urinary proteins for (**A**) Albumin, (**B**) Cystatin C, (**C**) KIM-1, (**D**) B2M, (**E**) RBP4, (**F**) FABP1, (**G**) EGF, (**H**) Clusterin, (**I**) TIMP1, and (**J**) IP10. Models shown were constrained in the positive direction with 100 repeated holdout validation, adjusted for urinary creatinine, socioeconomic status, child age, sex, smoking inside home, and body mass index *z*-score.

The WQS regression analysis of indices assessing significant associations with metal mixtures in urine to individual kidney injury biomarkers is shown in Table 3. For the urine metals mixture, per each metal quartile increase, urinary B2M was 0.18 ng/mL (95% CI: 0.05, 0.32) higher, attributable to the contribution of Cd (47%), Hg (23%), and As (19%). Per each quartile increase in the urine metal mixture, urinary albumin was 0.23 ng/mL (95% CI: 0.10, 0.37) higher, primarily due to the contributions of Cd (50%) and As (20%). We observed no significant associations between eGFR, NGAL, A1M, OPN, uromodulin, GSTα, calbindin, and renin, and either the blood or urine metal mixture indices. The urine metal weights derived for the multi-metal index in WQS with each individual urinary protein are shown in Figure 3. All of the associations with metal mixtures in urine and blood to individual kidney injury biomarkers assessed with the WQS regression analysis are presented in Supplemental Table S3.

## 4. Discussion

Overall, we found that prenatal urinary metal concentrations, both individually and as a mixture, were associated with altered urinary kidney injury biomarkers measured in healthy children. These associations were predominantly observed for second trimester urinary As and Cd and null associations were generally observed with blood metal concentrations.

Urinary As and Pb concentrations in this Mexico City population were similar to As (geometric mean: 15.21 μg/L), and higher, respectively, than urinary Pb (geometric mean: 0.92 μg/L) in a birth cohort in Greece, which reported associations between prenatal metal exposure and elevated blood pressure throughout childhood [47]. Second trimester metal concentrations in this population were higher than those reported in 1283 pregnant women enrolled in the National Health and Nutrition Examination Survey which reported Cd geometric mean blood levels of 0.27 mg/L and Pb blood levels of 0.62 μg/dL. One study of 909 healthy children aged 10–18 years in Sri Lanka reported urine concentrations of kidney injury biomarkers [48]. The concentrations reported in the Sri Lanka study (NGAL (2.86 ng/mL) and KIM-1 (0.11 ng/mL)) were lower than the median concentrations of NGAL (8.24 ng/mL) and KIM-1 (0.45 ng/mL) in our study [48]. There is limited information on reference levels of kidney injury biomarkers among healthy children, and often, adult reference data have been used to generalize pediatric reference intervals [49].

Evidence supports that both occupational and environmental exposure to individual metals and metalloids can lead to an increased risk of CKD and tubular indicators of dysfunction [15,20,22,50]. Exposure to Cd causes dysfunction of the proximal tubule in the kidney, which may result in increased urinary excretion of low-molecular-weight proteins, including A1M, B2M, and RBP4; as such, prior cross-sectional studies have reported associations of low-level Cd exposure with tubular indicators of kidney disease [51,52,53,54]. Among 222 healthy male sugarcane cutters in Guatemala, a repeated cross-sectional study conducted over one year reported that low urine Cd concentrations (median range: 0.09–0.14 μg/L) were associated with higher urine NGAL excretion, with the observed associations at Cd levels below those previously associated with renal injury in prior studies [55]. A study of 490 Chinese women aged 35–54 years reported that increasing levels of urinary Cd were significantly associated with markers of tubular renal effects, as indicated by increased urinary N-acetyl-beta-d-glucosaminidase (NAG) and B2M [56]. Similarly, we observed that a doubling of urine Cd was associated with higher urinary B2M as well as comprising 51% of the metal mixture weights contributing to the association for B2M. Several studies have reported that urinary B2M concentrations, measured alone or in combination with other glomerular and tubular analytes, can be used to detect Cd-induced renal dysfunction at early stages [57]. We also observed that a doubling of urine Cd was associated with higher albumin, A1M, and RBP4. These findings herein also suggest that renal tubules may be affected by prenatal exposure to Cd, even at low exposure levels. Additionally, we report that a doubling of urine Cd and Pb was associated with higher TIMP1. TIMP1 regulates extracellular matrix production and inhibits collagen degradation enzymes (which includes matrix-metallo-proteinases), resulting in the development of tubulointerstitial fibrosis and worsening inflammation [58,59]. These findings could be impactful for advancing the development of biomarkers for diagnosing CKD progression.

Our findings suggest an association between combined exposure to As, Cd, Hg, and Pb as measured in urine with kidney injury biomarkers that is in line with previous research, although to our knowledge no prior study has examined mixed prenatal metal exposures. In this study, urine Cd was weighted as the largest contributor to the metal mixture index in identified associations with six kidney injury biomarkers (albumin, B2M, RPB4, EGF, clusterin, and TIMP1). A mixture of As, Cd, Pb, and Hg levels measured in urine was associated cross-sectionally with higher eGFR and urine albumin levels in 12–19-year-old children in the United States [60]. In this prior study the association between the metals mixture and urine albumin was also driven by Cd (37%) in a similar proportion to what we have reported (50%) [60]. Another cross-sectional study among 1435 adults in the United States aged 40 years or older found that exposure to metal mixtures (e.g., cobalt, chromium, Cd, Hg, and Pb) in blood was associated with indicators of worse kidney function [61]. The findings included suggest that combined prenatal metal exposures may lead to subclinical glomerular or tubular damage assessed by urinary proteins in the absence of worse eGFR. While some metals such as As, Cd, and Hg are directly toxic to podocytes in glomeruli [62], acute exposure to toxicants including Cd, Pb, and Hg can also occur via reabsorption in the apical membrane of the first zone of the proximal tubule, in addition to the loop of Henle, altering ion transport pathways with potential direct cellular toxicity [20,63,64]. Both acute and chronic toxicity can inhibit mitochondrial respiration and initiate apoptotic signaling cascades via the generation of reactive oxygen species [63,65]. Chronic exposure to toxic metals can lead to oxidative stress (via the depletion of glutathione or impaired metallothionein detoxification pathways) and inflammation that influence the progression of CKD or renal failure [65,66]. Yet, knowledge gaps remain in the developmental effects of metal(loid)s on renal function and maturation processes, as well as the cumulative effects in early adulthood.

While traditional indicators of kidney function, including eGFR, serum creatinine, and albumin-to-creatinine ratio are used to diagnose acute kidney injury or CKD, newer biomarkers hold the potential of detecting renal damage at earlier stages. Urine biomarkers may be better able to predict renal function decline and CKD diagnosis than blood biomarkers, based on improved sensitivity and specificity [67], and urine collection is non-invasive, therefore more easily accessible in large population-based studies. The use of a single biomarker may not be ideal for predicting CKD progression because it may not fully characterize the complicated and compounded pathophysiological processes [68]. A multi-panel platform with biomarker specificity to nephron functional region (such as glomerular or specific tubular segments) may be more informative to determine critical sites of damage or treatment in renal insufficiency. Urinary proteomics or customized panels hold promise for biomarker discovery in this field [18].

Our study had a few limitations. Prenatal metal concentrations and preadolescent kidney injury proteins were assessed at a single time point. We selected second trimester metal measurements to examine our hypothesis of exposure during a sensitive window of renal development; future studies may examine longitudinal kidney outcomes. Our assessment of urine proteins was limited to those on three pre-established panels of acute kidney injury. As with any observational study, we cannot rule out residual or unmeasured confounding due to unmeasured factors that could influence both prenatal metal exposure and protein concentrations in childhood. The timing of urine sample collection was not systematically recorded; however, the majority of urine samples were collected in the morning of each visit. We also did not speciate As or Hg metabolites which can vary proportionally by exposure sources, such as diet or geographic factors [69,70]. Toxicokinetic differences in metal and metalloid distribution, metabolism, and excretion also influence the selection of metal biomatrix and measured concentrations [71]. In this study we did not specifically account for the source of metal or the route of absorption which varies by study population. Lastly, as we did not identify a priori a single kidney biomarker as a primary outcome variable, we conducted multiple statistical testing on multiple biomarkers which may have increased the risk of false positive findings in our analyses. Our study also had many strengths. Metals were assessed in samples collected prenatally which enabled an assessment of the longitudinal relationship with kidney injury biomarker outcomes, thus limiting reverse causation bias that was of concern in previous cross-sectional studies [72]. The participants in PROGRESS are generally healthy with no history of clinical renal disease; thus, we did not anticipate directionality to be confounded by disease status. However, since our study population included relatively healthy women and children, it may not be generalizable to populations with CKD. This study also assessed metals in two biomatrices (urine and blood), which enabled a comparison of observed differences specific to each medium. Along with important covariates, we adjusted for hydration status. We further applied WQS, an established mixtures method, which allowed for an assessment of the joint effect of multiple metals on kidney injury biomarkers. By employing the WQS method, we accounted for collinearity among multiple predictors, as well as allowing for the detection of multi-metal contributors to the association with kidney injury biomarkers [73].

## 5. Conclusions

We found that prenatal urinary metals were associated with urinary kidney injury biomarkers in healthy children in the PROGRESS longitudinal birth cohort study. Exposure to prenatal metals may lead to later subclinical glomerular or tubular injury in children with potential implications for susceptibility throughout the life course. Further studies are needed to examine nephrotoxicant exposure effects on subclinical kidney injury at later life stages, as well as to better understand metal mixtures and potential mechanisms of action in distinct nephron segments and transport processes during development that may affect kidney health in later life.

## Figures and Tables

**Table 1:** Demographic information and descriptive statistics for PROGRESS subjects (n = 494) in the study.

	N (%)
**Child Sex**	
Male	252 (51.01)
Female	242 (48.99)
**Socioeconomic Status during Pregnancy**	
Lower	265 (53.64)
Medium	184 (37.25)
Higher	45 (9.11)
**Child Body Mass Index**	
Normal	272 (55.06)
Overweight	118 (23.89)
Obese	104 (21.05)
**Indoor Tobacco Smoke Exposure during Pregnancy**	
No	344 (69.64)
Yes	150 (30.36)
	**Mean (Range)**
Age at urine collection (years)	9.66 (8.08–12.07)
Child Body Mass Index *z*-score	0.86 (−3.00–3.98)
**Kidney function measures**	
eGFR (mL/min/1.73 m^2^) (n = 422)	99.50 (46.76–201.33)
Serum Cystatin C (mg/L)	0.73 (0.32–1.56)
	**Median (25th–75th Percentile)**
**Second Trimester Urine Metal Concentrations ^†^**	
Arsenic (µg/L)	13.72 (9.07–22.48)
Cadmium (µg/L)	0.22 (0.14–0.37)
Lead (µg/L)	3.42 (2.08–6.66)
Mercury (µg/L)	1.12 (0.67–2.14)
**Second Trimester Blood Metal Concentrations (n = 470)**	
Arsenic (µg/dL)	0.07 (0.06–0.09)
Cadmium (µg/dL)	0.02 (0.02–0.03)
Lead (µg/dL)	2.85 (1.97–4.40)
**Urinary Kidney Injury Biomarkers at 8–12 years of age**	
Albumin (mg/dl)	2.39 (1.26–4.80)
Cystatin C (ng/mL)	12.03 (4.94–21.62)
KIM-1 (ng/mL)	0.45 (0.21–0.79)
NGAL (ng/mL)	8.24 (3.38–22.91)
A1M (ng/mL)	170.74 (108.53–265.76)
B2M (ng/mL)	223.43 (79.05–473.11)
RBP4 (ng/mL)	1418.52 (625.26–2813.50)
OPN (ng/mL)	774.13 (246.53–1404.00)
Uromodulin (MFI)	3943.00 (2476.73–5747.65)
GSTα (ng/mL)	0.67 (0.09–4.47)
FABP1 (ng/mL)	17.91 (12.98–26.47)
EGF (ng/mL)	43.13 (26.08–70.14)
Clusterin (ng/mL)	678.26 (341.57–1334.53)
Calbindin (ng/mL)	22.29 (8.26–66.17)
TIMP1 (ng/mL)	0.94 (0.63–1.49)
IP10 (ng/mL)	0.004 (0.003–0.02)
Renin (ng/mL)	0.08 (0.02–0.18)

^†^ Second trimester urine metal concentrations are specific gravity-normalized. eGFR: estimated glomerular filtration rate; KIM-1: kidney injury molecule-1; NGAL: neutrophil gelatinase-associated lipocalin; A1M: alpha-1-microglobulin; B2M: beta-2-microglobulin; RBP4: retinol-binding protein 4; OPN: osteopontin; MFI: mean fluorescence intensity; GSTα: glutathione S-transferase alpha; FABP1: fatty acid binding protein 1; TIMP1: TIMP metallopeptidase inhibitor 1; IP10: interferon gamma-induced protein 10.

**Table 2:** Linear regressions of second trimester blood and urine metals with eGFR and individual urinary proteins assessed at age 8–12 years.

	Urine								Blood					
	Arsenic		Cadmium		Mercury		Lead		Arsenic		Cadmium		Lead	
	Beta	95% CI	Beta	95% CI	Beta	95% CI	Beta	95% CI	Beta	95% CI	Beta	95% CI	Beta	95% CI
**Glomerular**														
eGFR (mL/min/1.73 m^2^)	−0.19	−1.78–1.40	−1.27	2.99–0.45	0.69	−0.89–2.27	0.27	−1.22–1.78	1.03	−2.19–4.24	−1.07	−3.89–1.75	−0.08	−2.46–2.30
Albumin (mg/dL)	**0.16**	**0.05–0.26**	**0.22**	**0.11–0.33**	0.10	−0.002–0.21	**0.14**	**0.04–0.24**	−0.04	−0.25–0.17	0.06	−0.12–0.23	0.02	−0.13–0.18
Cystatin C (ng/mL)	**0.11**	**0.01–0.21**	**0.13**	**0.03–0.23**	0.03	−0.06–0.13	0.08	−0.01–0.17	−0.06	−0.25–0.13	0.09	−0.07–0.25	0.03	−0.11–0.17
**Tubular**														
KIM-1 (ng/mL)	0.07	−0.01–0.16	0.08	−0.01–0.17	0.05	−0.04–0.13	**0.08**	**0.01–0.16**	−0.09	−0.26–0.08	0.11	−0.03–0.25	−0.01	−0.13–0.12
NGAL (ng/mL)	0.16	−0.08–0.41	0.16	−0.11–0.42	−0.03	−0.27–0.22	0.18	−0.05–0.42	−0.02	−0.53–0.49	0.18	−0.24–0.61	−0.07	−0.44–0.31
A1M (ng/mL)	0.02	−0.04–0.08	**0.08**	**0.02–0.14**	0.03	−0.03–0.08	0.02	−0.03–0.07	0.002	−0.11–0.12	0.04	−0.06–0.13	0.02	−0.07–0.10
B2M (ng/mL)	0.11	−0.01–0.23	**0.14**	**0.01–0.28**	0.07	−0.05–0.20	0.02	−0.10–0.14	**−0.25**	**−0.50–−0.001**	0.09	−0.12–0.30	−0.01	−0.20–0.17
RBP4 (ng/mL)	0.07	−0.04–0.17	0.10	−0.01–0.22	0.03	−0.08–0.14	0.03	−0.07–0.14	**−0.28**	**−0.49–−0.07**	0.12	−0.06–0.30	−0.03	−0.19–0.12
OPN (ng/mL)	−0.01	−0.14–0.11	0.01	−0.12–0.15	0.02	−0.10–0.15	0.05	−0.07–0.16	−0.12	−0.39–0.13	−0.12	−0.33–0.09	−0.03	−0.21–0.16
Uromodulin (MFI)	−0.002	−0.08–0.08	−0.01	−0.10–0.08	−0.03	−0.11–0.06	−0.05	−0.13–0.03	**−0.18**	**−0.34–−0.02**	0.02	−0.12–0.15	−0.09	−0.21–0.03
GSTα (ng/mL)	0.09	−0.14–0.31	0.12	−0.13–0.36	0.19	−0.03–0.41	0.03	−0.18–0.24	0.01	−0.44–0.46	**0.47**	**0.09–0.85**	0.06	−0.28–0.39
**Liver**														
FABP1 (ng/mL)	0.06	−0.003–0.13	0.04	−0.03–0.11	0.04	−0.02–0.11	0.02	−0.04–0.08	0.07	−0.06–0.20	−0.01	−0.12–0.10	−0.01	−0.10–0.09
**General**														
EGF (ng/mL)	0.03	−0.03–0.08	**0.07**	**0.01–0.12**	0.04	−0.02–0.09	0.03	−0.02–0.08	**−0.11**	**−0.22–−0.003**	0.02	−0.08–0.11	−0.02	−0.10–0.06
Clusterin (ng/mL)	0.06	−0.03–0.15	**0.12**	**0.03–0.22**	0.05	−0.04–0.14	0.06	−0.03–0.15	−0.09	−0.27–0.10	0.01	−0.14–0.16	0.02	−0.12–0.14
Calbindin (ng/mL)	−0.03	−0.20–0.14	0.07	−0.11–0.25	−0.01	−0.18–0.16	−0.09	−0.25–0.06	−0.03	−0.38–0.31	0.06	−0.24–0.35	−0.17	−0.42–0.08
TIMP1 (ng/mL)	**0.06**	**0.01–0.12**	**0.10**	**0.04–0.16**	0.03	−0.03–0.08	**0.06**	**0.005–0.11**	−0.06	−0.18–0.05	0.09	−0.01–0.18	0.005	−0.08–0.09
IP10 (ng/mL)	**0.13**	**0.02–0.24**	0.09	−0.03–0.21	−0.05	−0.17–0.06	0.02	−0.08–0.13	0.08	−0.15–0.30	−0.12	−0.31–0.07	0.02	−0.15–0.18
Renin (ng/mL)	0.08	−0.05–0.21	0.09	−0.04–0.23	−0.01	−0.14–0.12	0.01	−0.11–0.14	0.09	−0.17–0.35	−0.06	−0.29–0.16	0.02	−0.17–0.22

CI: confidence interval; eGFR: estimated glomerular filtration rate; KIM-1: kidney injury molecule-1; NGAL: neutrophil gelatinase-associated lipocalin; A1M: alpha-1-microglobulin; B2M: beta-2-microglobulin; RBP4: retinol-binding protein 4; OPN: osteopontin; MFI: mean fluorescence intensity; GSTα: glutathione S-transferase alpha; FABP1: fatty acid binding protein 1; EGF: epidermal growth factor; TIMP1: TIMP metallopeptidase inhibitor 1; IP10: interferon gamma-induced protein 10. Beta estimates and 95% CIs in bold indicate *p* < 0.05.

**Table 3:** Associations of urine multi-metal index analyses ^†^ with individual urinary proteins ^‡^ and derived metal weights.

	n	Estimate	Standard Error	2.50%	97.50%	Metal Weights			
						w1	w2	w3	w4
**Glomerular**									
Albumin (ng/mL)	491	0.23	0.07	0.10	0.37	Cd: 0.50	As: 0.20	Hg: 0.15	Pb: 0.15
Cystatin C (ng/mL)	494	0.17	0.07	0.05	0.31	As: 0.36	Cd: 0.32	Hg: 0.17	Pb: 0.14
**Tubular**									
KIM-1 (ng/mL)	494	0.13	0.05	0.02	0.24	As: 0.31	Pb: 0.27	Hg: 0.23	Cd: 0.20
B2M (ng/mL)	493	0.18	0.07	0.05	0.32	Cd: 0.47	Hg: 0.23	As: 0.19	Pb: 0.11
RBP4 (ng/mL)	494	0.15	0.07	0.02	0.28	Cd: 0.51	Pb: 0.19	Hg: 0.15	As: 0.14
**Liver**									
FABP1 (ng/mL)	494	0.11	0.04	0.03	0.19	As: 0.69	Hg: 0.16	Cd: 0.13	Pb: 0.02
**General**									
EGF (ng/mL)	494	0.06	0.03	0.002	0.13	Cd: 0.45	Hg: 0.25	Pb: 0.18	As: 0.13
Clusterin (ng/mL)	494	0.13	0.05	0.03	0.22	Cd: 0.47	Pb: 0.22	As: 0.17	Hg: 0.13
TIMP1 (ng/mL)	494	0.13	0.04	0.05	0.20	Cd: 0.44	As: 0.30	Hg: 0.14	Pb: 0.12
IP10 (ng/mL)	494	0.15	0.07	0.02	0.28	As: 0.65	Cd: 0.21	Pb: 0.10	Hg: 0.03

^†^ The associations presented in this table were selected from the weighted quantile regression analyses. Full weighted quantile regression analyses are shown in Supplemental Table S3. ^‡^ Models shown were constrained in the positive direction with 100 repeated holdout validation, adjusted for urinary creatinine, socioeconomic status, child age, sex, smoking inside home, and body mass index *z*-score. KIM-1: kidney injury molecule-1; NGAL: neutrophil gelatinase-associated lipocalin; A1M: alpha-1-microglobulin; FABP1: fatty acid binding protein 1; TIMP1: TIMP metallopeptidase inhibitor 1; IP10: interferon gamma-induced protein 10.

## Data Availability

The data that were used in this study can be made accessible to researchers upon appropriate request with restrictions to ensure the privacy of human subjects. Note that access to the data is limited due to a data sharing agreement approved by the IRB at Mount Sinai.

## Supplementary Materials

The following supporting information can be downloaded at: https://www.mdpi.com/article/10.3390/toxics10110692/s1, Table S1: Distribution of creatinine-normalized urinary kidney injury biomarker levels (ng/mg creatinine) measured at 8–12 years of age; Table S2: Linear regressions of individual second trimester blood and urine metals (quartiles) and kidney injury biomarkers (log_2_ transformed) for comparison with WQS models; Table S3: Associations of urine and blood multi-metal index analyses with individual urinary proteins and derived metal weights. Figure S1. Pearson correlation matrix of second trimester blood and urine metals and kidney injury biomarkers (log_2_ transformed) for comparison.
