# Supplementary material for: Prenatal Metal Exposures and Associations with Kidney Injury Biomarkers in Children
Source: Toxics. 2022 Nov 16;10(11):692. doi: 10.3390/toxics10110692 (PMC9699100; doi:10.3390/toxics10110692)
Supplement: Supplementary file 1 [file toxics-10-00692-s001.zip › toxics-1996397-supplementary.pdf]

1. Supplemental Materials

1.1. Supplemental Results

As a secondary analysis, results of single metal linear regression models with metal concentrations as quartiles and log_2 transformed kidney injury biomarker concentrations for comparison with results of WQS regression models, are shown in Supplemental Table 2. A quartile increase of urine As was associated with a 0.13 ng/ml (95% CI: 0.01, 0.30) higher FABP1 and 0.12 ng/ml (95% CI: 0.02, 0.21) higher KIM-1, with similar specific pairwise metal associations of urine As with albumin, cystatin C, TIMP1, and IP10 as above. We observed similar specific pairwise metal associations of urine Cd with proteins including albumin, cystatin C, A1M, B2M, EGF, clusterin, and TIMP1 as above, however the relationship with NGAL was null and a positive relationship was now identified with RBP4 (0.17 ng/ml; 95% CI: 0.05, 0.29). We observed significant associations between urine Hg with GTSα, which was null in the primary models with continuous metals levels as the predictor. The association between urine Pb and TIMP1 was also null. We observed similar specific pairwise metal associations of blood As with B2M, RBP4, and EGF, and blood Cd with GSTα.

Supplemental Table S1. Distribution of creatinine-normalized urinary kidney injury biomarker levels (ng/mg creatinine) measured at 8-12 years of age.†

Urinary kidney injury biomarker	Median (Interquartile Range)
Albumin	23.27 (14.67-51.07)
Cystatin C	12.24 (6.63-19.43)
KIM-1	0.47 (0.27-0.76)
NGAL	9.19 (3.36-24.63)
A1M	180.50 (132.29-253.60)
B2M	259.35 (92.32-483.53)
RBP4	1511.70 (705.13-2569.97)
OPN	852.31 (296.97-1351.42)
GSTα	0.68 (0.11-5.64)
FABP1	20.13 (13.11-32.04)
EGF	45.36 (33.98-59.54)
Clusterin	762.17 (474.52-1263.30)
Calbindin	24.24 (10.21-59.34)
TIMP1	1.06 (0.78-1.42)
IP10	0.01 (0.004-0.02)
Renin	0.06 (0.03-0.20)

† Uromodulin was excluded from this table because the value was in mean fluorescence intensity units. KIM-1: kidney injury molecule-1; NGAL: neutrophil gelatinase-associated lipocalin; A1M: alpha-1-microglobulin; B2M: beta-2-microglobulin; RBP4: retinol-binding protein 4; OPN: osteopontin; GSTα: glutathione S-transferase alpha; FABP1: fatty acid binding protein 1; EGF: epidermal growth factor; TIMP1: TIMP metallopeptidase inhibitor 1; IP10: interferon gamma-induced protein 10.

Supplemental Figure S1. Pearson correlation matrix of second trimester blood and urine metals and kidney injury biomarkers (log_2 transformed) for comparison.

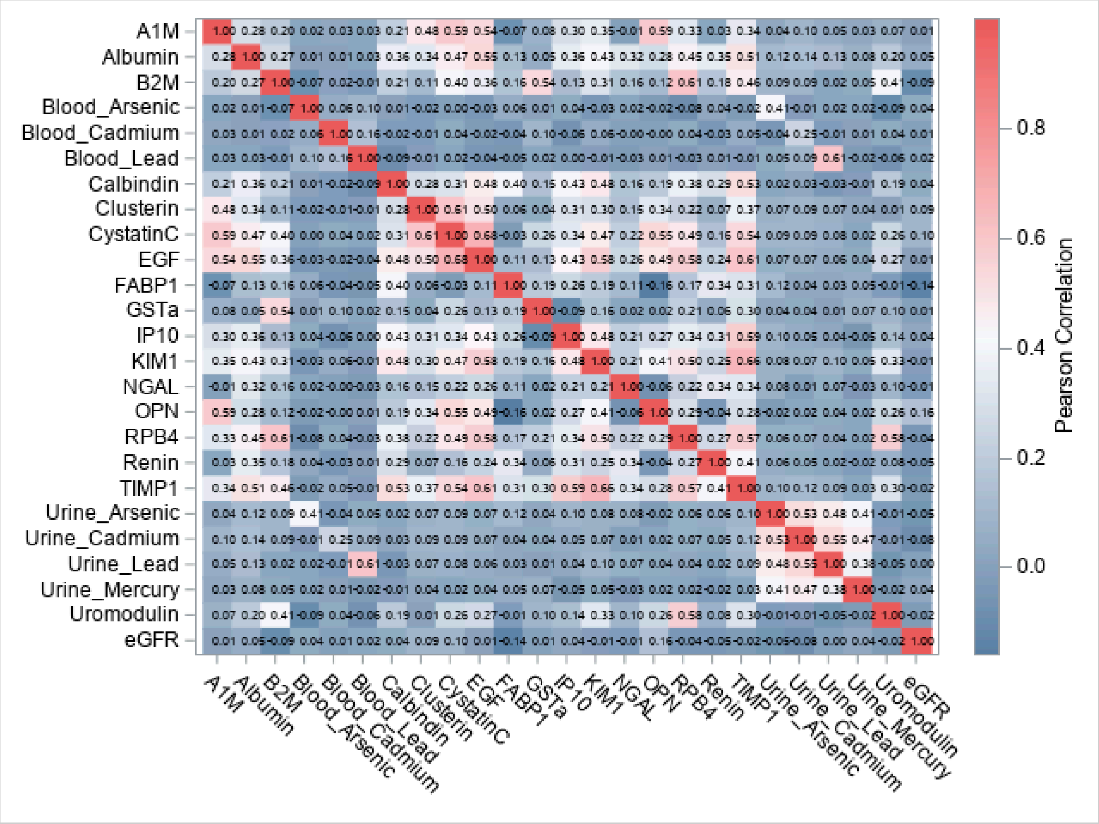

A1M: alpha-1-microglobulin; B2M: beta-2-microglobulin; EGF: epidermal growth factor; FABP1: fatty acid binding protein 1; GSTα: glutathione S-transferase alpha; IP10: interferon gamma-induced protein 10; KIM-1: kidney injury molecule-1; NGAL: neutrophil gelatinase-associated lipocalin; OPN: osteopontin; RBP4: retinol-binding protein 4; TIMP1: TIMP metallopeptidase inhibitor 1; eGFR: estimated glomerular filtration rate.

Supplemental Table S2. Linear regressions of individual second trimester blood and urine metals (quartiles) and kidney injury biomarkers (log_2 transformed) for comparison with WQS models.

	Urine								Blood					
	Arsenic		Cadmium		Mercury		Lead		Arsenic		Cadmium		Lead	
	Beta	95% CI	Beta	95% CI	Beta	95% CI	Beta	95% CI	Beta	95% CI	Beta	95% CI	Beta	95% CI
**Glomerular**														
eGFR (mL/min/1.73 m^2)	-0.22	-2.06-1.62	-0.95	-2.81-0.90	0.53	-1.30-2.37	0.88	-0.97-2.72	1.30	-0.61-3.22	-0.48	-2.43-1.47	0.13	-1.77-2.05
Albumin (mg/dl)	**0.13**	**0.01-0.25**	**0.21**	**0.09-0.33**	0.09	-0.03-0.21	**0.13**	**0.01-0.25**	-0.02	-0.14-0.10	0.05	-0.07-0.18	0.01	-0.11-0.14
Cystatin C (ng/ml)	**0.15**	**0.040.26**	**0.14**	**0.03-0.25**	0.08	-0.03-0.19	0.09	-0.02-0.20	-0.02	-0.13-0.09	0.08	-0.03-0.19	0.02	-0.09-0.14
**Tubular**														
KIM-1 (ng/ml)	**0.12**	**0.02-0.21**	0.09	-0.002-0.19	0.07	-0.03-0.17	**0.10**	**0.004-0.20**	-0.05	-0.15-0.05	0.07	-0.03-0.17	0.01	-0.09-0.11
NGAL (ng/ml)	0.23	-0.05-0.51	0.24	-0.04-0.52	0.03	-0.25-0.31	0.21	-0.08-0.49	0.10	-0.20-0.39	0.18	-0.12-0.48	0.04	-0.26-0.34
A1M (ng/ml)	0.03	-0.04-0.09	**0.08**	**0.01-0.15**	0.03	-0.04-0.10	0.02	-0.05-0.09	-0.02	-0.09-0.05	0.04	-0.03-0.10	0.01	-0.06-0.08
B2M (ng/ml)	0.10	-0.04-0.25	**0.19**	**0.05-0.33**	0.11	-0.04-0.25	0.06	-0.08-0.20	**-0.17**	**-0.31--0.02**	0.04	-0.11-0.19	0.001	-0.15-0.15
RBP4 (ng/ml)	0.08	-0.05-0.20	**0.17**	**0.05-0.29**	0.05	-0.07-0.17	0.08	-0.04-0.20	**-0.16**	**-0.29--0.04**	0.08	-0.05-0.20	-0.001	-0.13-0.12
OPN (ng/ml)	0.01	-0.14-0.15	-0.01	-0.15-0.13	0.04	-0.10-0.19	0.06	-0.08-0.20	-0.03	-0.18-0.11	-0.07	-0.22-0.07	-0.03	-0.18-0.12
Uromodulin (MFI)	0.004	-0.09-0.10	0.01	-0.07-0.10	0.02	-0.07-0.11	-0.03	-0.12-0.07	**-0.11**	**-0.20--0.01**	0.01	-0.08-0.11	-0.05	-0.14-0.04
GSTα (ng/ml)	0.20	-0.06-0.45	0.20	-0.05-0.45	**0.27**	**0.02-0.53**	0.02	-0.24-0.27	-0.04	-0.30-0.23	**0.31**	**0.05-0.58**	0.06	-0.20-0.33

**Liver**																
FABP1 (ng/ml)	**0.13**	**0.05-0.20**	0.05	-0.02-0.13	0.04	-0.04-0.12	-0.01	-0.08-0.07	0.03	-0.05-0.10	0.01	-0.07-0.09	0.0004	-0.08-0.08
**General**																
EGF (ng/ml)	0.02	-0.04-0.08	**0.08**	**0.02-0.14**	0.04	-0.02-0.10	0.03	-0.03-0.09	**-0.06**	**-0.13--0.001**	0.01	-0.05-0.08	0.002	-0.06-0.07
Clusterin (ng/ml)	0.07	-0.03-0.18	**0.14**	**0.04-0.25**	0.04	-0.06-0.15	0.09	-0.02-0.19	-0.04	-0.15-0.06	0.02	-0.08-0.13	0.02	-0.09-0.12
Calbindin (ng/ml)	-0.01	-0.21-0.18	0.05	-0.14-0.25	0.003	-0.19-0.20	-0.07	-0.27-0.12	-0.04	-0.24-0.16	0.06	-0.15-0.26	-0.09	-0.29-0.11
TIMP1 (ng/ml)	**0.09**	**0.03-0.16**	**0.11**	**0.05-0.18**	0.05	-0.01-0.11	0.06	-0.01-0.12	-0.05	-0.11-0.02	0.07	-0.0001-0.13	0.02	-0.05-0.08
IP10 (ng/ml)	**0.17**	**0.04-0.29**	0.08	-0.05-0.21	-0.06	-0.18-0.07	0.02	-0.10-0.15	0.05	-0.08-0.18	-0.06	-0.19-0.07	0.04	-0.09-0.18
Renin (ng/ml)	**0.15**	**0.01-0.30**	0.10	-0.05-0.25	-0.06	-0.21-0.09	-0.02	-0.17-0.13	0.05	-0.11-0.20	-0.03	-0.19-0.12	-0.01	-0.16-0.15

eGFR: estimated glomerular filtration rate; KIM-1: kidney injury molecule-1; NGAL: neutrophil gelatinase-associated lipocalin; A1M: alpha-1-microglobulin; B2M: beta-2-microglobulin; RBP4: retinol-binding protein 4; OPN: osteopontin; MFI: mean fluorescence intensity; GSTα: glutathione S-transferase alpha; FABP1: fatty acid binding protein 1; EGF: epidermal growth factor; TIMP1: TIMP metallopeptidase inhibitor 1; IP10: interferon gamma-induced protein 10. Beta estimates and 95% CIs in bold indicate $p<0.05$.

Supplemental Table S3. Associations of urine and blood multi-metal index analyses† with individual urinary proteins‡ and derived metal weights. Linear regressions of individual second trimester blood and urine metals (quartiles) and kidney injury biomarkers (log_2 transformed) for comparison with WQS models.

	n	Estimate	Standard Error	2.50%	97.50%	Metal weights			
						w1	w2	w3	w4
**Urine Multi-Metal Index**									
**Glomerular**									
eGFR (mL/min/1.73 m^2)	422	0.09	0.93	-1.73	1.91	Pb: 0.46	Hg: 0.32	As: 0.16	Cd: 0.06
Albumin (ng/ml)	491	0.23	0.07	0.10	0.37	Cd: 0.50	As: 0.20	Pb: 0.15	Hg: 0.15
Cystatin C (ng/ml)	494	0.17	0.07	0.04	0.31	As: 0.36	Cd: 0.32	Hg: 0.17	Pb: 0.14
**Tubular**									
KIM-1 (ng/ml)	494	0.13	0.05	0.02	0.24	As: 0.31	Pb: 0.27	Hg: 0.23	Cd: 0.20
NGAL (ng/ml)	493	0.19	0.13	-0.07	0.45	Cd: 0.33	Pb: 0.28	As: 0.27	Hg: 0.12
A1M (ng/ml)	494	0.07	0.04	-0.01	0.14	Cd: 0.51	Hg: 0.19	As: 0.17	Pb: 0.13
B2M (ng/ml)	493	0.18	0.07	0.05	0.32	Cd: 0.47	Hg: 0.23	As: 0.19	Pb: 0.11
RBP4 (ng/ml)	494	0.15	0.07	0.02	0.28	Cd: 0.51	Pb: 0.19	Hg: 0.15	As: 0.14
OPN (ng/ml)	493	0.02	0.07	-0.12	0.15	Pb: 0.36	Hg: 0.35	As: 0.16	Cd: 0.13
Uromodulin (MFI)	494	-0.02	0.05	-0.13	0.08	Cd: 0.31	Hg: 0.31	As: 0.24	Pb: 0.14
GSTα (ng/ml)	494	0.25	0.14	-0.03	0.53	Hg: 0.38	Cd: 0.28	As: 0.26	Pb: 0.08
**Liver**									
FABP1 (ng/ml)	494	0.11	0.04	0.03	0.19	As: 0.69	Hg: 0.16	Cd: 0.13	Pb: 0.02
**General**									
EGF (ng/ml)	494	0.06	0.03	0.00	0.13	Cd: 0.45	Hg: 0.25	Pb: 0.18	As: 0.13
Clusterin (ng/ml)	494	0.13	0.05	0.03	0.22	Cd: 0.47	Pb: 0.22	As: 0.17	Hg: 0.13
Calbindin (ng/ml)	494	-0.04	0.10	-0.24	0.16	Cd: 0.41	Hg: 0.30	As: 0.17	Pb: 0.12
TIMP1 (ng/ml)	494	0.13	0.04	0.05	0.20	Cd: 0.44	As: 0.30	Hg: 0.14	Pb: 0.12
IP10 (ng/ml)	494	0.15	0.07	0.02	0.28	As: 0.65	Cd: 0.21	Pb: 0.10	Hg: 0.03
Renin (ng/ml)	494	0.11	0.08	-0.04	0.27	As: 0.57	Cd: 0.29	Hg: 0.07	Pb: 0.07
**Blood Multi-Metal Index**									
**Glomerular**									

eGFR (mL/min/1.73 m²)	406	0.45	1.10	-1.71	2.60	As: 0.55	Pb: 0.29	Cd: 0.17	-
Albumin (ng/ml)	467	-0.01	0.07	-0.14	0.13	Cd: 0.51	Pb: 0.26	As: 0.22	-
Cystatin C (ng/ml)	470	0.05	0.07	-0.09	0.18	Cd: 0.54	Pb: 0.26	As: 0.20	-
**Tubular**									
KIM-1 (ng/ml)	470	0.03	0.06	-0.09	0.16	Cd: 0.60	Pb: 0.29	As: 0.12	-
NGAL (ng/ml)	469	0.11	0.17	-0.22	0.43	Cd: 0.40	As: 0.32	Pb: 0.28	-
A1M (ng/ml)	470	0.01	0.04	-0.07	0.09	Cd: 0.53	Pb: 0.30	As: 0.17	-
B2M (ng/ml)	469	-0.02	0.08	-0.18	0.15	Cd: 0.57	Pb: 0.37	As: 0.06	-
RBP4 (ng/ml)	470	0.04	0.08	-0.11	0.19	Cd: 0.64	Pb: 0.33	As: 0.04	-
OPN (ng/ml)	469	-0.12	0.08	-0.28	0.04	Pb: 0.39	As: 0.33	Cd: 0.28	-
Uromodulin (MFI)	470	-0.03	0.06	-0.15	0.09	Cd: 0.69	Pb: 0.24	As: 0.07	-
GSTα (ng/ml)	470	0.25	0.14	-0.02	0.53	Cd: 0.63	Pb: 0.21	As: 0.16	-
**Liver**									
FABP1 (ng/ml)	470	0.01	0.04	-0.07	0.08	As: 0.43	Cd: 0.30	Pb: 0.27	-
**General**									
EGF (ng/ml)	470	-0.02	0.04	-0.09	0.06	Cd: 0.50	Pb: 0.38	As: 0.12	-
Clusterin (ng/ml)	470	-0.04	0.07	-0.17	0.10	Cd: 0.44	Pb: 0.40	As: 0.16	-
Calbindin (ng/ml)	470	-0.07	0.12	-0.30	0.16	Cd: 0.52	As: 0.26	Pb: 0.21	-
TIMP1 (ng/ml)	470	0.05	0.04	-0.02	0.13	Cd: 0.65	Pb: 0.28	As: 0.07	-
IP10 (ng/ml)	470	0.02	0.08	-0.14	0.17	As: 0.46	Pb: 0.39	Cd: 0.14	-
Renin (ng/ml)	470	-0.03	0.09	-0.20	0.14	As: 0.46	Cd: 0.27	Pb: 0.27	-

[†] Models shown were constrained in the positive direction with 100 repeated holdout validation, adjusted for urinary creatinine, socioeconomic status, child age, sex, smoking inside home, and body mass index z-score.

eGFR: estimated glomerular filtration rate; KIM-1: kidney injury molecule-1; NGAL: neutrophil gelatinase-associated lipocalin; A1M: alpha-1-microglobulin; B2M: beta-2-microglobulin; RBP4: retinol-binding protein 4; OPN: osteopontin; MFI: mean fluorescence intensity; GSTα: glutathione S-transferase alpha; FABP1: fatty acid binding protein 1; EGF: epidermal growth factor; TIMP1: TIMP metallopeptidase inhibitor 1; IP10: interferon gamma-induced protein 10.
